# Supplementary material for: Evaluation of a Maternal Plasma RNA Panel Predicting Spontaneous Preterm Birth and Its Expansion to the Prediction of Preeclampsia
Source: Diagnostics (Basel). 2022 May 27;12(6):1327. doi: 10.3390/diagnostics12061327 (PMC9221694; doi:10.3390/diagnostics12061327)
Supplement: Supplementary file 1 [file diagnostics-12-01327-s001.zip › diagnostics-1731012-supplementary.pdf]

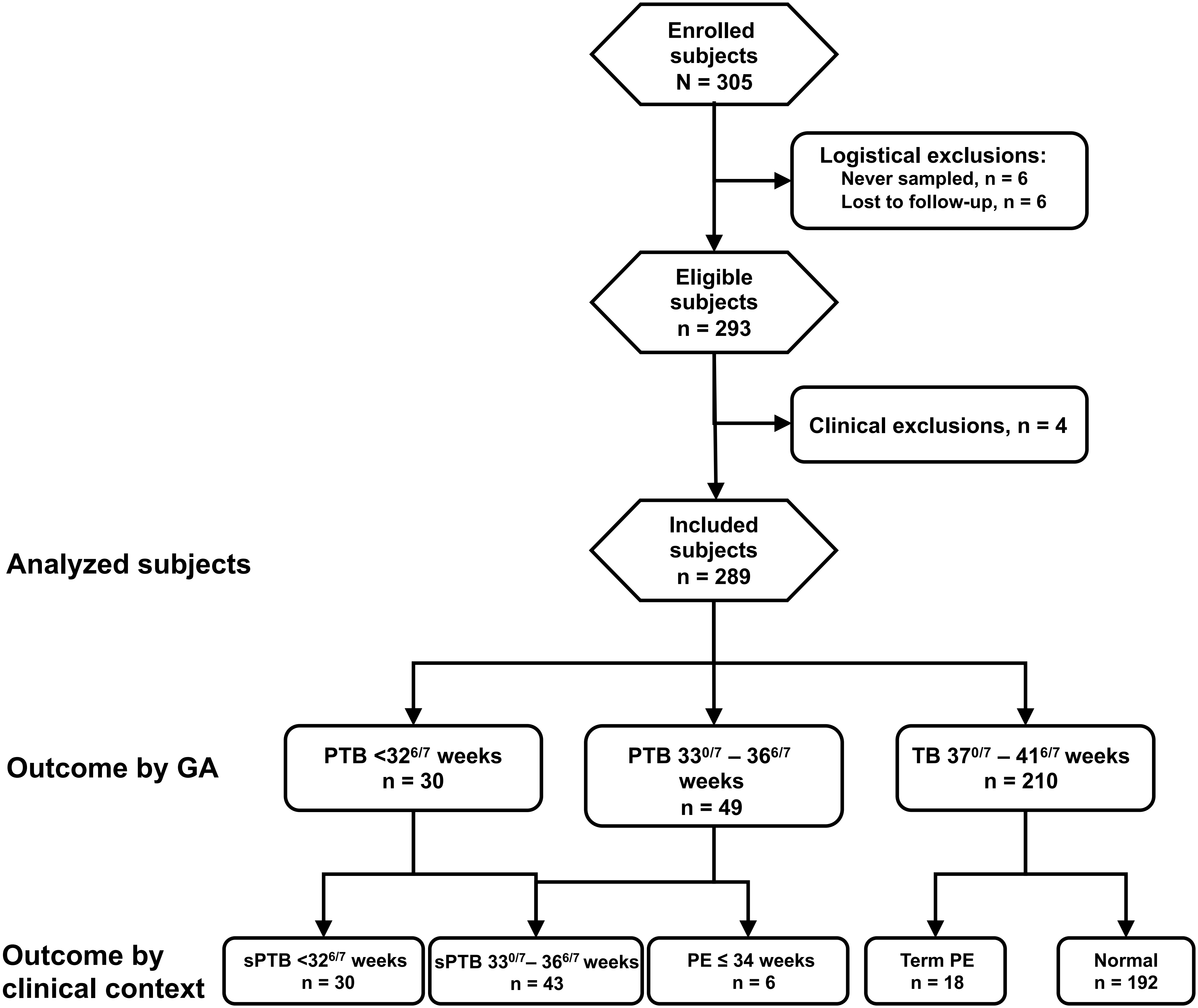

Supplemental Figure 1. STARD flow diagram for a study of 289 patients undergoing FutureBIRTH™ screening between 16-19- 5/7 weeks. Legend- TB: term birth; PTB: preterm birth; sPTB: spontaneous preterm birth; PE: preeclampsia
